# Relationship between manual dexterity and left–right asymmetry of anatomical and functional properties of corticofugal tracts revealed by T2-weighted brain images

**DOI:** 10.1038/s41598-023-29557-1

**Published:** 2023-02-15

**Authors:** Noriyuki Oka, Masaharu Sakoh, Misato Hirayama, Mayu Niiyama, Albert Gjedde

**Affiliations:** 1 Convalescent Rehabilitation Center, Nerima Ken-Ikukai Hospital, 7-3-28, Ooizumigakuen-chou, Nerima-ku, Tokyo, 178-0061 Japan; 2 Department of Nuclear Medicine and PET Center, Aarhus University Hospital, Palle Juul-Jensens Boulevard 99, 8200 Aarhus N, Denmark; 3 Translational Neuropsychiatry Unit, Department of Clinical Medicine, Aarhus University, Universitetsbyen 13, Building 2B, 8000 Aarhus C, Denmark; 4 Department of Neuroscience, University of Copenhagen, 3 Blegdamsvej, 2200 Copenhagen, Denmark; 5 McConnell Brain Imaging Center, Montreal Neurological Institute, Department of Neurology and Neurosurgery, McGill University, 3801 Rue University, Montreal, QC H3A 2B4 Canada

**Keywords:** Brain, Motor control

## Abstract

The corticofugal tracts (CFT) are key agents of upper limb motor function. Although the tracts form high-intensity regions relative to surrounding tissue in T2-weighted magnetic resonance images (T2WI), the precise relations of signal intensities of the left and right CFT regions to hand function are unknown. Here, we tested the hypothesis that the different signal intensities between the left and right CFT signify clinically important differences of hand motor function. Eleven right-handed and eleven left-handed healthy volunteers participated in the study. Based on horizontal T2WI estimates, we confirmed the relationship between the signal intensity ratios of the peak values of each CFT in the posterior limbs of the internal capsules (right CFT vs. left CFT). The ratios included the asymmetry indices of the hand motor functions, including grip and pinch strength, as well as the target test (TT) that expressed the speed and accuracy of hitting a target ([right-hand score − left-hand score]/[right-hand score + left-hand score]), using simple linear regression. The signal intensity ratios of each CFT structure maintained significant linear relations with the asymmetry index of the speed (R^2^ = 0.493, P = 0.0003) and accuracy (R^2^ = 0.348, P = 0.004) of the TT. We found no significant association between left and right CFT structures for grip or pinch strengths. The findings are consistent with the hypothesis that the different signal intensities of the left and right CFT images captured by T2WI serve as biological markers that reflect the dominance of manual dexterity.

## Introduction

Lateralization of brain function is a major but incompletely understood feature of human brain organization^1,2^. In the service of this organization, the dual corticofugal tract (CFT) pathways including the corticospinal tracts form major lateralized groups of nerve fibers that serve motor functions of the left and right extremities^3^. The CFT fibers are visible as high-intensity structures on T2-weighted images (T2WI) recorded by magnetic resonance imaging (MRI)^4–6^. In clinical practice, adjustment of the contrast of T2WI greatly facilitates the visualization of the CFT bundles by reference to the different intensities of the left and right CFT signals. However, it is a problem that the relationship between the intensity of CFT signals and motor function remains undefined. In addition, the origin of the high-intensity regions is also unclear in the T2WI, as the high-signal intensity may reflect protons. It is also possible that the high-intensity signal in the posterior limb of the internal capsule is related to absence of ferritin^7^. Therefore, we needed to test whether the high-signal intensity is related to motor function, as already confirmed for the fractional anisotropy (FA) signal.

Hervé et al.^8^ determined the difference between the intensities of left and right CFT signals from the posterior limbs of the internal capsules of right- and left-handed healthy volunteers, using an index of apparent gray matter density. The results showed higher apparent gray matter density of the left than of the right CFT of right-handed participants, unlike the apparent gray matter densities of left-handed participants that had greater symmetry. The report suggested that the different intensities of left and right CFT signals depend on the lateralization of the dominant hand, but it remains unknown to which extent measures of hand motor function specifically relate to the signal intensities of left and right CFT fibers.

Hand motor properties include functions related to muscle force, including grip and pinch strengths, as well as functions related to manual dexterity that requires fine coordination of muscle contractions. The hand motor functions depend on brain activity that arises in the frontal lobes, in part as the result of conscious decisions^9^, and changes of manual dexterity and grip and pinch strengths accompany morphological changes of the CFT structures. In previous studies, Jang et al.^10^ reported that “walnut-rolling” with the non-dominant hand improved the ability to manipulate a peg, raised grip and pinch strengths, and significantly increased the apparent number of fibers in contralateral CFT of the non-dominant hand of healthy participants. Jang and Seo^11^ reported that pinch strength and peg manipulation measures increased, and the contralateral apparent CFT volume rose significantly after two weeks of peripheral stimulation of the extensor digitorum muscle of healthy volunteers. Jang and Jang^12^ had shown that the apparent number of fibers of the CFT of the uninjured hemisphere increased when dominant hand lateralization changed after stroke. From these findings, we conclude that the asymmetry of signal intensities of the left and right CFT images reflects structural differences of CFT fibers that we claim underlie the asymmetry of manual dexterity and muscle strength measures.

Angstmann et al.^13^ reported a linear relationship between the differences of left- and right hand dexterity measures during a circle-drawing task as well as asymmetry of FA values of the left and right CFT fibers in the posterior limbs of the internal capsules of right-handed volunteers. The FA values of diffusion tensor imaging represent objective measures of CFT integrity, but they are not easily used in normal clinical settings because of the need for special software. Instead, T2-weighted imaging is a routine application of software to the interpretation of CFT signals that reflect specific hand functions. This means that CFT signals from the right hand reflect functions that we associate with the right hand according to a questionnaire. Results from T2-weighted imaging therefore directly reflect the magnitude and progress of skill learning tasks.

Here, we tested the hypothesis that the type of hand function (i.e., muscle force or manual dexterity) determines the signal intensity differences of the left and right CFT signals recorded by T2WI in right- and left-handed participants with different hand functions.

## Materials and methods

### Participants

Twenty-two healthy volunteers participated in the study. We recruited eleven right-handed participants (6 men and 5 women; 25.3 ± 3.5 years old; FLANDERS handedness score^14^: 9.8 ± 0.4) and eleven left-handed participants (6 men and 5 women; 25.9 ± 3.2 years old; FLANDERS handedness score: − 9.1 ± 1.4), with ages and sexes matched as well as possible. All participants declared that they had no history of central nervous system disease or psychiatric disorders.

### Acquisition of brain imaging data and measurement of signal intensity of bilateral CFT

With 1.5-Tesla MRI (Siemens Healthcare K.K., MEGNETOM ESSENZA, A Tim and Dot, Japan), we acquired horizontal T2WI (6-mm slice thickness, 1.2 mm gaps, echo time [TE] = 98 ms, repetition time [TR] = 4000 ms, base resolution = 448, voxel size = 0.5 × 0.5 × 6.0 mm^3^, field of view = 230 × 230 mm^2^, flip angle = 170°, and bandwidth = 130 Hz/pixel). We acquired all images in parallel to the anterior and posterior commissures^15^ and confirmed the absence of significant lateral tilt to the images by the symmetries of structures along the axis passing through the anterior and posterior commissures (e.g., eyeball, mammillary body, optic chiasm, middle cranial fossa edge, and auditory canal).

**Figure 1:**
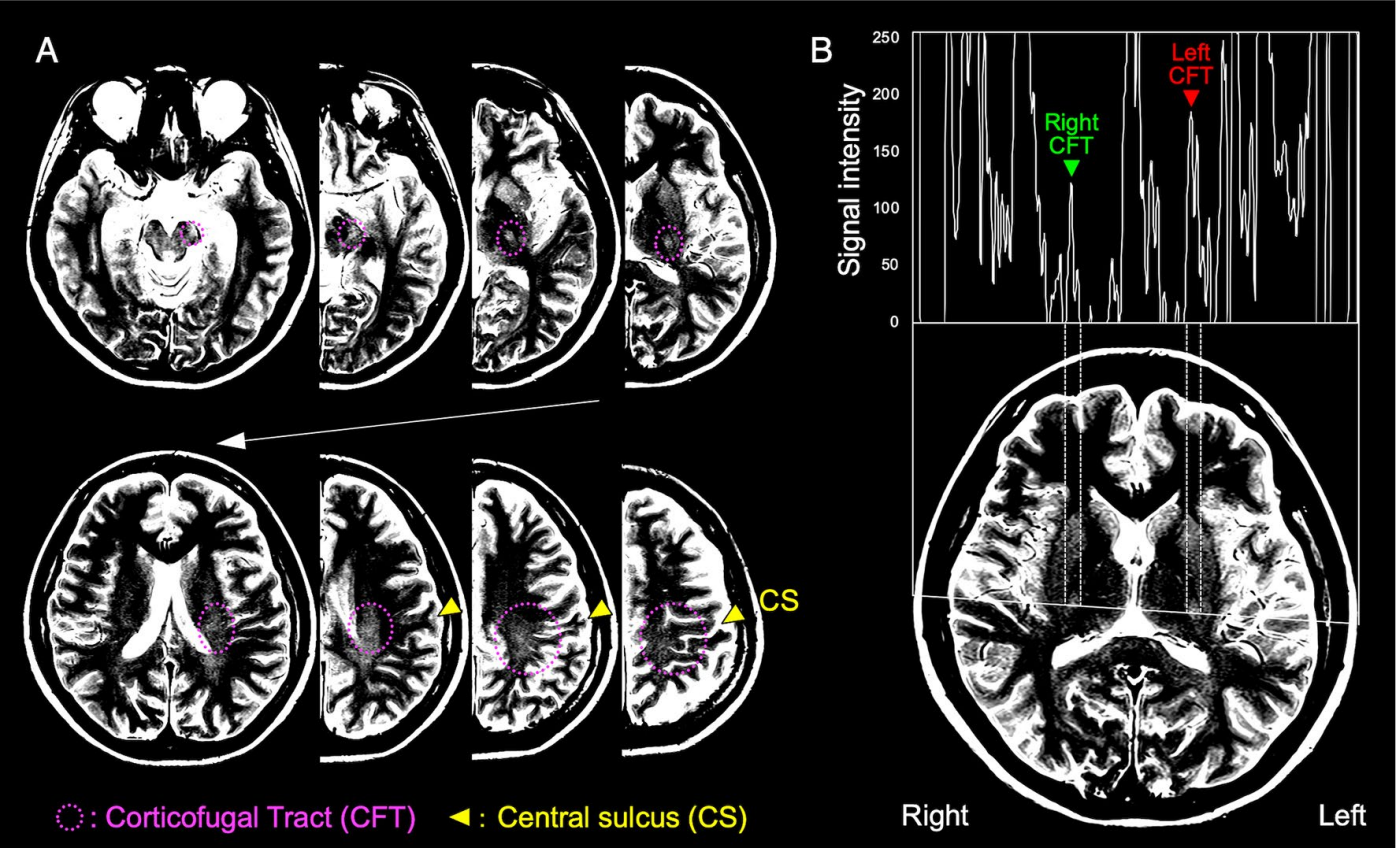
Peak signal intensity of bilateral CFT structures. (**A**) The area identified as CFT had higher signal intensity than the surrounding tissue and was continuous from the cerebral peduncle to the motor related cortex. (**B**) We measured the peak signal intensity values of the CFT structures by plotting the signal intensity of the line segments passing through the center of the bilateral CFT structures as a histogram.

To measure the signal intensities of bilateral CFT structures of the T2WI, we selected an image that included the fornix column at one horizontal slice above the image passing through the anterior and posterior commissures, and we identified the locations of the bilateral CFT structures within the posterior limbs of the internal capsule in the contrast-adjusted image. To improve the visualization of the CFT by T2WI, we adjusted the contrast of the images by setting the window-wide value to 100 and the window-level value to 5 increments (window level = 377.7 ± 20.9) using the DICOM Workstation and Viewer (Miele-LXIV), within which the structures of the CFT, capsule, and globus pallidus could be seen separately (Fig. 1A). In the contrast-adjusted T2WI as an eight-bit gray-scale image, we detected the luminance distribution of the pixels by the image-processing software ImageJ (National Institutes of Health, USA), at the line segment passing through the center of the left and right CFT elements. We adopted the peak value of the luminance of each CFT structure as the measured value (Fig. 1B). The same examiner obtained the measurements three times, and we used the mean values for analysis.

### Hand motor function data acquisition

#### Grip and pinch strengths

**Figure 2:**
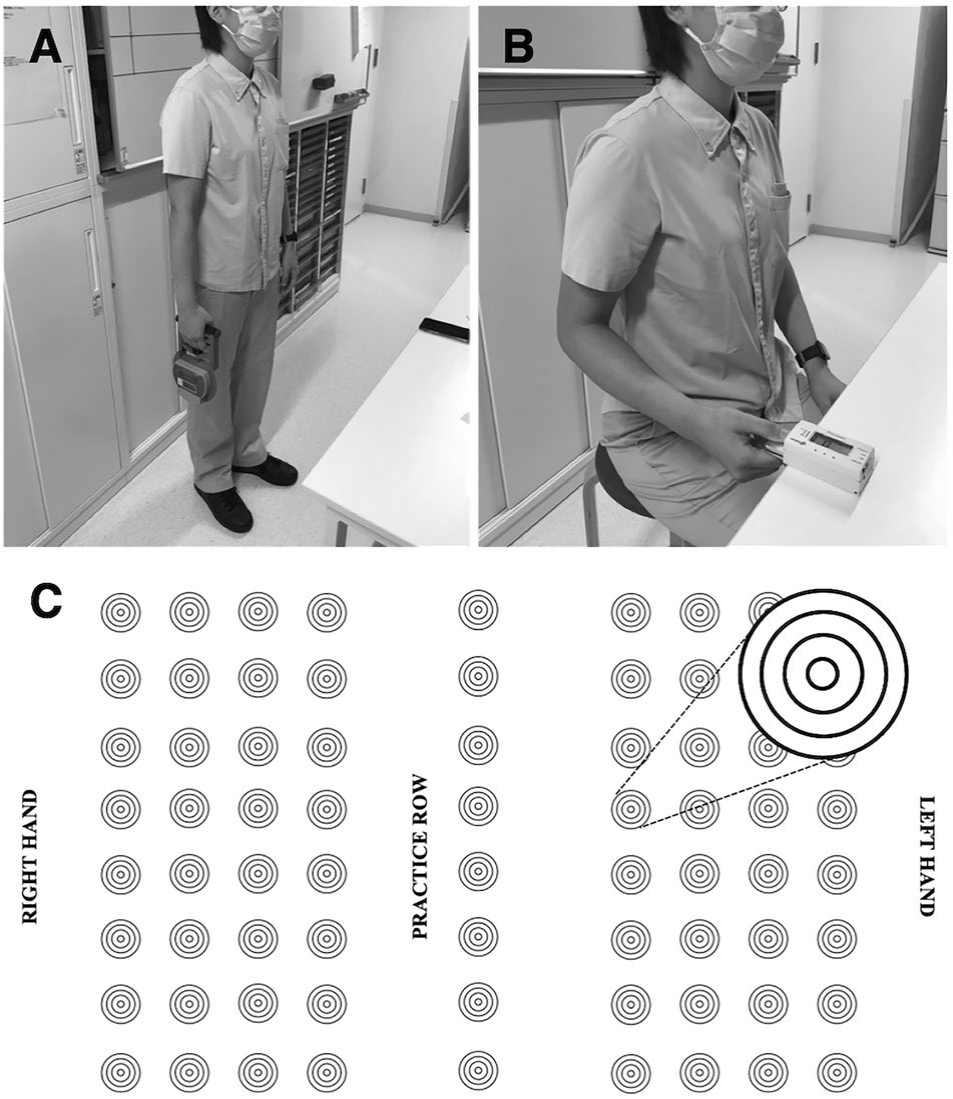
Measurement of hand motor performance. (**A**) Grip strength measurement. (**B**) Lateral pinch strength measurement. (**C**) Paper format of the Target Test.

We measured grip strengths of both hands as directed by the Japanese Physical Fitness Test Guidelines^16^ with a Smedley-type digital grip dynamometer (Grip-D, Takei Scientific Instruments Co., Ltd., Japan) (Fig. 2A). We adjusted the grip width to place the proximal interphalangeal joint of the index finger approximately at 90° during the grasping of the grip dynamometer. To measure grip strength, we asked the volunteers to lower both arms naturally, taking care not to let the grip dynamometer touch the body or clothing. We measured grip strengths twice, alternating the right and left sides and adopting the higher values for analysis. We measured lateral pinch strength with a pinch dynamometer (MT-100, SAKAI Medical Co., Ltd., Japan) (Fig. 2B). We obtained the measurements with the radial surface between the distal and proximal interphalangeal joints of the index finger and thumb, with the shoulder joint in 0° flexion, abduction, and internal/external rotation, the elbow joint in 90° flexion, and the forearm in the middle position. We obtained two measurements, alternating each side, and adopting the higher value for analysis.

#### Hand dexterity

To evaluate manual dexterity, we used a procedure based on a study of the Target Test (TT)^17^, designed to prepare and administer the test with the left and right hands (Fig. 2C). We administered the test with pencil and paper on which four targets were printed horizontally and eight vertically (the smallest circle 2 mm in diameter, and the largest a quadrupled circle with a diameter of 12 mm). The test has two components, a speed test and an accuracy test. We evaluated the speed test as the time taken to mark all of the smallest targets (circles with a diameter of 2 mm), judging shorter times of completion to reflect greater dexterity. For the accuracy test, subjects aimed at one of the smallest circles and marked all targets at once, with a 144-Hz rhythm. Higher scores reflected marks closer to the center of the target, and higher accuracy reflected higher total scores of all targets, with a maximum score of 256 points.

### Data analysis

In each analysis, we tested normality of the data by means of the Shapiro–Wilk test that did not confirm normality for some groups. For this reason, we chose non-parametric testing. Thus, for the CFT structures, for each dominant hand, we compared the signal intensities of the left and right CFT structures by Mann–Whitney *U* testing. We calculated peak signal intensity as the ratio of results of the left to right hemispheres (right/left). For hand motor function scores, grip and pinch strengths, and scores of speed and accuracy, we calculated test scores of TT as an asymmetry index ([right − left]/[left + right]). We did a second analysis to confirm whether every index reflected the effect of handedness, and we compared the right- and left-handed groups by Mann–Whitney *U* testing. The magnitude of the effect size (r) was named “small” at 0.1 or greater, “medium” at 0.3 or greater, and “large” at 0.5 or greater^18^. To confirm the relationship between the signal intensity values of the CFT structures and the hand motor functions, we did a simple linear regression of the asymmetry indices of hand motor function against the signal intensity ratios of the CFT structures as the independent variables as a third analysis, and we did the same for each gender separately as a fourth analysis. We used SPSS Statistic 29.0 (IBM, Japan) for the analysis with the significance level set at less than 5%.

### Ethics approval

This study was conducted in accordance with the principles of the Declaration of Helsinki, as approved by the Ethics Review Committee of Nerima Ken-ikukai Hospital Medical Corporation (Ethics #10).

### Consent to participate and publish

All participants gave verbal and written informed consents to the use and publication of anonymized data.

## Results

### Comparison of the signal intensities of right and left CFT in each dominant hand group

**Figure 3:**
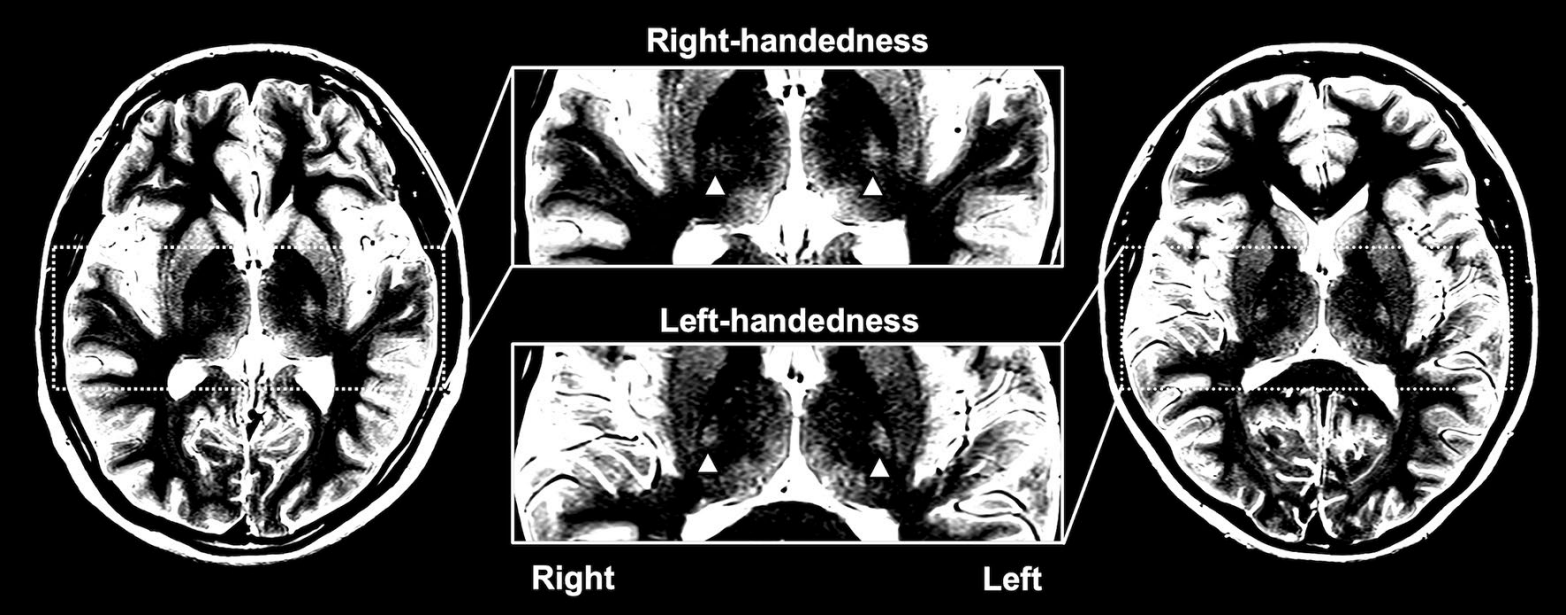
Bilateral corticofugal fibers signal intensities in right- and left-handed individuals (typical cases). The brain image on the left shows the slice of a right-handed participant, and the brain image on the right shows the slice of a brain image of a left-handed participant (window level 375, window width 100). White triangles indicate the CFT structures. For right-handed participants, the signal intensity of the left CFT structure was higher than that of the right CFT structure. For left-handed participants, we found the signal intensities of the left and right CFT structures to be almost identical.

The signal intensities of right-handed volunteers exceeded those of left-handed volunteers, with left-handed participants having less asymmetry. Figure 3 shows the difference between the left and right CST signal intensities for representative right- and left-handed participants. In the right-handed groups, the signal intensity of the left CFT significantly exceeded that of the right CFT (P = 0.013). In contrast, there was no significant difference of signal intensities between the right and left CFT of the members of the left-handed group (P > 0.05). Table 1 shows the median (25th and 75th percentiles) of signal intensities of the right and left CFT of each dominance group.

**Table 1:** Median differences in signal intensity of right and left CFT in each handedness.

	Right CFT [25th percentile, 75th percentile]	Left CFT [25th percentile, 75th percentile]	*P* value	Effect size (*r*)
Right-handedness (n = 11)	113.4 [108.8, 130.4]	141.1 [136.5, 171.5]	0.013*	0.733 [Large]
Left-handedness (n = 11)	144.7 [136.0, 160.4]	152.9 [137.6, 155.4]	1.000	0.010 [Negligible small]

*P < 0.05.

### Comparison of the right- and left-handed groups

We show the medians (25th and 75th percentiles) of the signal intensity ratios of the CFT images, the medians of the asymmetry indices of the grip and pinch strengths, and the values of speed and accuracy of the TT tests in Table 2. The signal intensity ratio of the CFT images differed significantly between the right- and left-handed groups (P < 0.05). The medians (25th and 75th percentiles) of the differences of grip strengths of the right and left hands (right − left) were 3.8 (0.1, 5.0) kg for the right-handed group and − 1.2 (− 2.4, 1.6) kg for the left-handed group. Regardless of hand dominance, the grip strength of the right hand was higher than that of the left hand, and the asymmetry indices of grip strengths significantly differed between the right- and left-handed groups (P < 0.05). The median of pinch strength differences between the right and left hands (right − left) were 1.1 (0.2, 1.5) kg for the right-handed group and − 0.5 (− 1.0, − 0.4) kg for the left-handed group, and higher for the dominant hand. This means that the asymmetry indices of pinch strengths differed significantly between the right- and left-handed groups (P < 0.05). The median differences of the speed tests of TT of right and left hands (right − left) were − 14.3 (− 18.8, − 10.2) seconds for the right-handed group and 6.6 (5.0, 10.7) seconds for the left-handed group, indicating faster completion by the dominant hand. Again, the asymmetry indices of the speed test of TT differed significantly between the groups (P < 0.05). Similarly, the median differences of accuracy by the TT tests between the right- and left-hand groups (right − left) were 35.0 (25.0, 40.5) points for the right-handed group, and − 22.0 (− 26.5, − 3.0) points for the left-handed group, with the dominant hand having a higher score. Therefore, the asymmetry indices of TT (accuracy) differed significantly between the right-handed and left-handed groups (P < 0.05).

**Table 2:** The signal ratio of the CFT and the asymmetry index of hand motor function.

		Right-handedness [25th percentile, 75th percentile] (n = 11)	Left-handedness [25th percentile, 75th percentile] (n = 11)	*P* value	Effect size (*r*)
CFT^a^	Signal intensity ratio	0.84 [0.76, 0.87]	1.02 [0.97, 1.09]	0.0001**	0.750 [Large]
Hand motor function^b^	Grip strength	0.04 [0.00, 0.07]	− 0.02 [− 0.04, 0.02]	0.0233*	0.477 [Medium]
	Pinch strength	0.07 [0.01, 0.09]	− 0.04 [− 0.06, − 0.02]	0.0004**	0.711 [Large]
	Target test (speed)	− 0.20 [− 0.23, − 0.16]	0.10 [0.07, 0.12]	< 0.0001**	0.848 [Large]
	Target test (accuracy)	0.08 [0.06, 0.10]	− 0.06 [− 0.06, − 0.01]	< 0.0001**	0.850 [Large]

^a^Ratio index is calculated as right CFT / left CFT.

^b^Asymmetry index of hand motor performance = (right score − left score)/(right score + left score).

*P < 0.05, **P < 0.01.

### Relationship between signal intensity ratios of the CFT measures and hand motor functions

**Figure 4:**
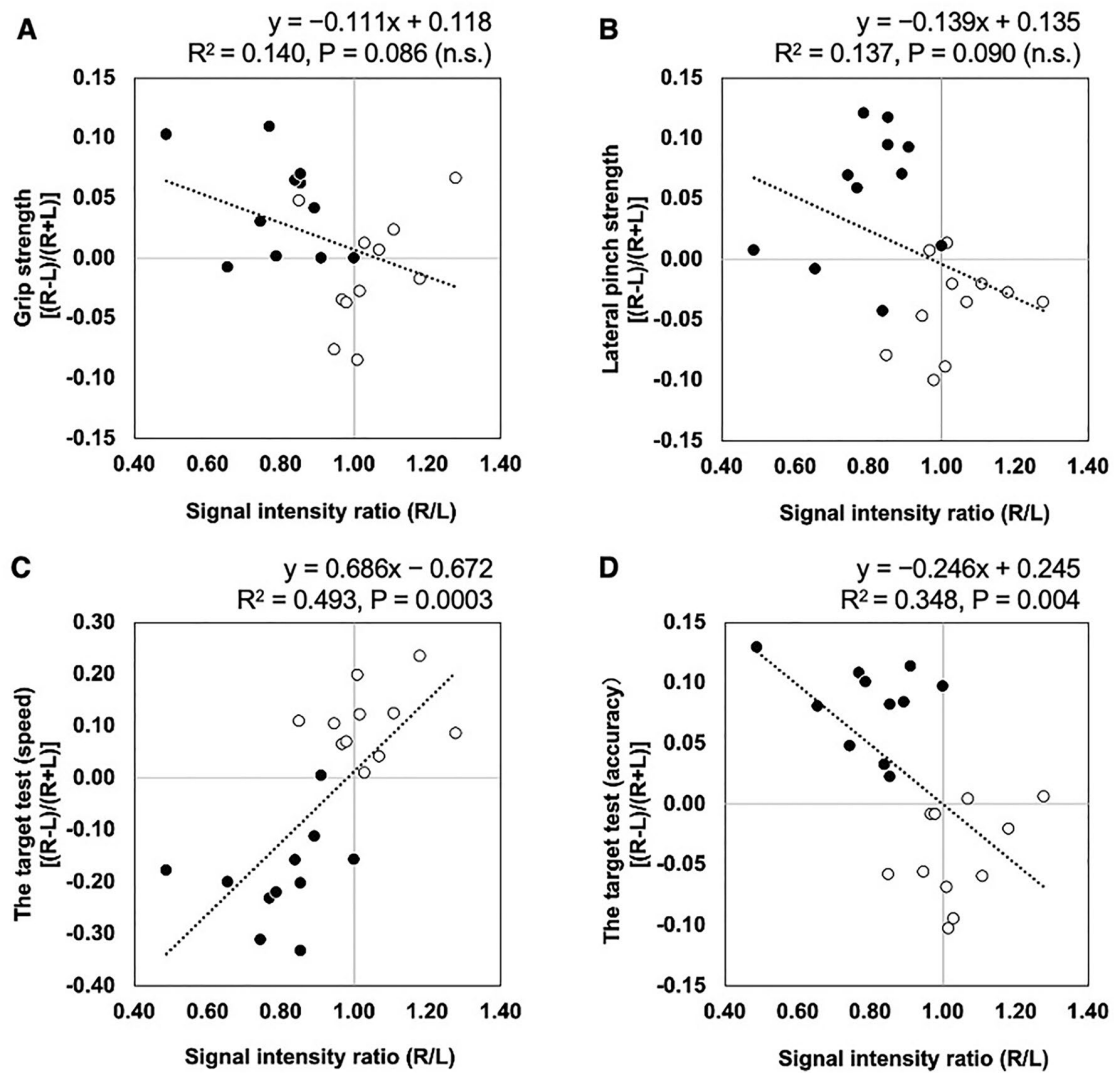
Relations among signal intensity ratios of CFT structures and hand motor performance asymmetry indices. (**A**) Grasp strength. (**B**) Lateral pinch strength. (**C**) Target test (speed test). (**D**) Target test (accuracy test). Black circles indicate right-handed participants. White circles indicate left-handed participants. *n.s.* non-significant.

Figure 4 shows the results of the simple linear regressions of the signal intensity ratio of the CFT images versus the asymmetry index of motor functions of each hand. The signal intensity ratio of the CFT images bore no relation to the asymmetry indices of grip and pinch strengths (grip strength, coefficient of determination [R^2^] = 0.140, P = 0.086; pinch strength R^2^ = 0.137, P = 0.090). In contrast, the asymmetry indices of the TT had significant association with the values of the signal intensity ratio of the CFT images in both speed and accuracy tests (speed test R^2^ = 0.493, P = 0.0003; accuracy test R^2^ = 0.348, P = 0.004).

### Differences of each indicator between right- and left-handed individuals according to gender and its relation to the signal intensity ratio of the CFT

Here, we present the median (25th and 75th percentiles) that results from the comparison of each index by gender in terms of handedness, as well as the results of the linear relationship between the asymmetry index and the signal intensity ratio of the left and right CFT structures for each motor performance.

The signal intensity ratio of the left and right CFTs showed a similar trend for both genders. In the men, the median of the right-handed participants was 0.814 (0.774, 0.879) and for the left-handed participants 1.069 (0.982, 1.163), significantly different at P = 0.015 (effect size r = 0.693 [large]). In the women, the median of the right-handed participants was 0.854 (0.745, 0.854) and for the left-handed participants 1.009 (0.978, 1.015), also significantly different at P = 0.007 (effect size r = 0.828[large]).

Grip strength asymmetry indices showed different trends of the genders. The index was 0.021 (0.0003, 0.059) for the right-handed men and 0.018 (− 0.010, 0.042) for the left-handed men, showing no significant difference (P = 0.699, effect size r = 0.139). The effect may relate to the fact that right hand grip strength may be stronger than left hand grip strength in males, even when judged by questionnaire to be left-handed. The association between grip strength asymmetry index and the signal intensity ratio of the left and right CFT structures was not significant in men (y = − 0.023x + 0.046, P = 0.760, R^2^ = 0.010). In contrast, the results for women were significantly different (P = 0.016, effect size r = 0.762), with 0.062 (0.030, 0.070) for right-handed participants and − 0.037 (− 0.076, − 0.027) for left-handed participants. Grip strength asymmetry index and the signal intensity ratio of the left and right CFT structures were significantly related (y = − 0.267x + 0.240, P = 0.016, R^2^ = 0.536). In other words, the stronger the left CFT signal when compared to the right CFT signal, the stronger the grip strength of the right hand was, compared to the left hand.

Pinch strength asymmetry index showed the same trend between genders. The pinch strength asymmetry index was 0.035 (− 0.003, 0.068) for right-handed men and − 0.024 (− 0.033, − 0.020) for left-handed men, with a significant difference (P = 0.065, effect size r = 0.558 [large]). The differences between right- and left-handed women were 0.092 (0.069, 0.095) and − 0.047 (− 0.089, − 0.035), respectively (P = 0.008, effect size r = 0.798 [large]). Pinch strength asymmetry index and the signal intensity ratio of the left and right CFT structures also were not significantly related in either gender (men: y = − 0.118x + 0.114, P = 0.218, R^2^ = 0.148; women: y = − 0.189x + 0.179, P = 0.244, R^2^ = 0.165).

The asymmetry indices of the TT (speed) showed the same trend of difference between the genders. The right-handed men had values of − 0.179 (− 0.215, − 0.158) and left-handed men values of 0.098 (0.071, 0.122), showing a significant difference (P = 0.002, effect size r = 0.833 [large]). The medians of right-handed and left-handed women were − 0.203 (− 0.311, − 0.178) and 0.105 (0.070, 0.123), respectively (P = 0.008, effect size r = 0.826 [large]), indicating a significant difference (P = 0.008, effect size r = 0.826 [large]). The relationship between the asymmetry index of the TT (speed) and the signal intensity ratio of the left and right CFT was significant for both genders (men: y = 0.677x − 0.677, P = 0.004, R^2^ = 0.578; women: y = 0.755x − 0.719, P = 0.031, R^2^ = 0.459).

The asymmetry index of TT (accuracy) showed the same trend of difference between genders. In men, the median of the right-handed participants was 0.090 (0.081, 0.100) and of the left-handed participants was − 0.039 (− 0.059, − 0.011), showing a significant difference (P = 0.002, effect size r = 0.837 [large]). In women, the median of the right-handed participants was 0.082 (0.048, 0.114) and of the left-handed participants − 0.056 (− 0.069, − 0.008), showing a significant difference (P = 0.008, effect size r = 0.826 [large]). The relationship between the asymmetry indices of the TT (accuracy) and the signal intensity ratio of the left and right CFT was significant only for women (men: y = − 0.202x + 0.214, P = 0.085, R^2^ = 0.268; women: y = − 0.332x + 0.309, P = 0.017, R^2^ = 0.527).

## Discussion

We quantified signal intensities of the CFT images obtained by T2WI of right- and left-handed healthy volunteers, and we tested the relation to hand motor functions. The results showed that the signal intensity ratios of the left and right CFT images differed according to handedness. We confirmed that the signal intensity of CFT fibers served as an index of motor function, because higher signal intensities of the left CFT, compared to the right CFT, matched the greater manual dexterities of the right hand, compared to the left hand.

### Signal intensities of left and right CFT structures depend on dominant hand

The signal intensity ratios of the CFT measures differed significantly between right- and left-handed participants. In right-handed participants, we identified higher signal intensities of left and right CFT structures in left than in right CFT, in contrast to the difference of signal intensities between left and right CFT images of left-handed participants that tended to be infinitesimally small. Previous studies reported the greater asymmetry of findings related to cerebral morphology of right-handed compared to left-handed subjects. Thus, Hervé et al.^8^ used T1-weighted images (T1WI) to gauge the signal intensity of the CFT at the posterior limb of the internal capsule as an index of apparent gray matter density. Also, Amunts et al.^19^ evaluated the use of the intrasulcal length of the posterior bank of the precentral gyrus (i.e., the depth of the central sulcus) measured by T1WI in horizontal sections as an index of asymmetry. In the present study, we confirmed the hypothetical claim of asymmetrical characteristics of a structure such as the CFT in relation to the dominant hand use of healthy volunteers.

In right-handed participants, the higher signal intensities of the left CFT than of the right CFT region may arise both from neurodevelopmental and from environmental factors. The CFT includes the corticospinal tract (CST), and the corticospinal tract of both sides have lower numbers of myelinated axons per unit area (myelin density) than the adjacent corticopontine tract and corticofugal fibers. This difference commonly is held to explain why the magnitudes of the signals from the corticospinal tract exceed the magnitudes of the signals from surrounding tissues^3^, because the thickness of the axons is likely to affect the myelin densities of both corticospinal tract structures. In fact, the corticospinal tract structures of both sides have larger axon diameters than any other region of the cerebrum^20^, and the thickening of axons by myelination during neuronal development contributes to increases of neuronal conduction velocities^21,22^. In other words, the CFT responsible for the dominant hand movements has both more mature and thicker axons and lower myelin density that contribute to the higher signal intensity. In addition, the nerve fibers contributing to the high signal intensity of the T2WI presumably are under strong influence by the corticospinal tract. The hand preference of most people determines the environment designed to accommodate the use of right hands^23^. This means that left-handed individuals often use the non-dominant right hand and hence have weaker laterality than right-handed individuals. The resulting activations of the fibers of both CFT pathways explain the lateralization of signal intensities of right-handed individuals to left CFT fibers, as well as the reduced difference of luminance between the right and left CFT structures of left-handed individuals.

### Relationship between signal intensity ratio of the left and right CFT structures and hand dexterity

While the hand motor functions showed better performance with the dominant hand, the signal intensity ratios of the CFT structures maintained a linear relationship between the results of the speed and accuracy tests of TT that reflect manual dexterity. The finding suggests that cases of higher signal intensity of left CFT compared to right CFT represent participants that completed the TT more rapidly with the right than with the left hand, and who more accurately marked the center of the target. In contrast, the signal intensity ratios of the CFT fibers did not reveal a significant linear relationship with the asymmetry indices of grip and pinch strengths. Therefore, the luminance ratios of the CFT structures are indices that reflect manual dexterity rather than hand force. In higher primates, including humans, axons of corticospinal tract fibers connect directly to the spinal motor neurons and play an important role in the control of fine hand movement^24–27^. Muir et al.^28^ reported that some cortical motor neurons of monkeys undergo activation during precise grip but not during power grip. In humans following stroke, hand movement and grasping abilities recovered after stroke-induced corticospinal tract injury, although the control of manual dexterity remained impaired^29^. The reports are consistent with the present study of CFT fibers that contribute to manual dexterity. The posterior limb of the internal capsule passes through the fibers from primary motor cortex (Brodmann area 4), the premotor cortex (Brodmann 6), the sensory cortex (Brodmann areas 3, 1, and 2), and adjacent parietal cortex (Brodmann area 5)^30^. Therefore, in addition to sensory function, the functions of the premotor cortex and parietal cortex^31^, which are considered to be involved in eye-hand movement necessary for manual dexterity, may also be factors that influence the results of this study.

### The effect of gender difference in signal intensity ratio and hand motor performance

Although the sample size was small, we also conducted a gender-specific analysis as an exploratory study. Here, the CST signal strength ratio, the TT (speed) and the TT (accuracy) asymmetry indices showed a common result of significantly superior performance of the dominant hand side for both genders. The linear relationship between the CST signal strength ratio and the asymmetric index of the TT (speed) and the asymmetric index of the TT (accuracy) showed generally the same trend as in the analysis of all participants. Therefore, we considered the relationship between the left–right ratio of CFT signal intensity and manual dexterity to be minimally affected by gender. However, we cannot ignore that the linear relationship between the asymmetry index of the TT (accuracy) and the ratio of the CST signal intensity for men was P < 0.1. Therefore, further studies with a larger sample size are necessary.

### Limitations and further study

In this study, we captured the continuity of the cerebral peduncle, the posterior limb of the internal capsule, and the motor cortex that form the ROIs of CST in other studies, consistent with the claim that the capture represents the CST. However, in order to fully identify the CST, it is important to determine that pyramidal crossing occurs in the medulla oblongata. To clarify this point, we need further studies of tractography.

One limitation is the effect of aging. As the results of the present study apply to adolescents, it is necessary to consider the effects of aging. In late adulthood, right–left asymmetry of corticospinal tract microstructures measured as FA value did not show a significant linear relationship with manual dexterity during circle drawing^32^. Furthermore, aging individuals may experience neurodegeneration associated with white matter lesions, at grade 2 or higher on the Fazekas Scale^33^, that may make elevated signals from T2WI of the left and right CFT difficult to distinguish from elevated signals generated by aging. In patients with amyotrophic lateral sclerosis, the luminance of CST fibers by T2WI is similar to that of cerebrospinal fluid due to demyelination, while the signal intensity of CST fibers of healthy people is said to be similar to that of the cortex^5^. Therefore, depending on the contrast adjustment of the images, the distinction may be uncertain and the methodology may need revision when the distinction is difficult between normal and abnormal images.

Structural changes of CFT fibers associated with the acquisition of motor skills can be confirmed by means of the FA value as an index^10–13^, but not by means of signal intensity changes of the CFT fibers yet. In clinical practice, the ataxia due to a cerebellar lesion, abnormal muscle tone, or a sensory abnormality due to a basal ganglia lesion, will present as motor disabilities, also when the CFT is not affected. Also, the state of the cerebellum activation that makes contributions to motor accuracy due to motor learning, may also affect the CFT activation. Myelination may be promoted by neural activity associated with functional activity^34^. Therefore, the involvement of other parts of the motor circuit, such as cerebellum or basal ganglia, that may be connected to the CFT, may change the signal intensity of the CFT. In an animal model of cerebral infarction, we showed that we could use the magnitude of the apparent diffusion coefficient as evidence against infarction after reperfusion^35,36^. It is uncertain whether the changes of the CFT signal reflect motor function during recovery from cerebral infarction, impairment of movement, or the effects of motor learning, but verification by longitudinal studies would lower the uncertainty, making it possible to raise the usefulness of luminance estimates of CFT structures as biomarker values that reflect motor function. The health of cerebral white matter is affected by multiple factors, including axonal packing density, axonal caliber, myelination, microglia, and tissue structure^37–39^. The activation sensitive signal arising from diffusion MRI of glia is of interest as a marker in this context^40^. It may be of relevance to further test the reproducibility and reliability of the imaging and the CFT signal strength measurements by application of complementary multimodal imaging techniques. Finally, in this study, the larger slice thickness (6 mm) at 1.5 T potentially may have affected the signal intensity of ROI measures.

## Conclusion

The findings may contribute to the assessment of motor function by brain imaging using routine T2WI, in addition to the traditional diagnosis of local brain injury.

## Data Availability

The datasets generated during or analyzed during the current study are not publicly available because the repository has not been set up but are available from the corresponding author on reasonable request.
